# Murine immune responses to a *Plasmodium vivax*-derived chimeric recombinant protein expressed in *Brassica napus*

**DOI:** 10.1186/1475-2875-10-106

**Published:** 2011-04-29

**Authors:** Choonghee Lee, Hyung-Hwan Kim, Kyung Mi Choi, Kyung Won Chung, Yien Kyoung Choi, Mi Jung Jang, Tong-Soo Kim, Nam-Jun Chung, Ho-Gun Rhie, Ho-Sa Lee, Youngjoo Sohn, Hyuck Kim, Sung-Jae Lee, Hyeong-Woo Lee

**Affiliations:** 1 Institute of Global Environment and Department of Biology, Kyung Hee University, Seoul 130-701, Republic of Korea; 2 Vascular Medicine Research Unit, Brigham and Women's Hospital, Harvard Medical School, Cambridge, MA 02139, USA; 3 Division of Malaria and Parasitic Diseases, National Institute of Health, Korea Centers for Disease Control and Prevention, Cheongwon-gun 363-951, Republic of Korea; 4 Department of Parasitology, College of Medicine, Inha University, Incheon 405-751, Republic of Korea; 5 Institute of Biological Resources, Callus Co., Ltd. Gwangju 500-420, Republic of Korea; 6 Department of Gynecology, College of Oriental Medicine, Sangji University, Wonju 220-717, Republic of Korea; 7 International Research Center for Bioscience and Biotechnology, Jungwon University, Goesan 367-805, Republic of Korea; 8 Department of Pathology, University of Florida, J-566, 1600 SW Archer Road, Gainesville, FL 32610, USA

## Abstract

**Background:**

To develop a plant-based vaccine against *Plasmodium vivax*, two *P. vivax *candidate proteins were chosen. First, the merozoite surface protein-1 (MSP-1), a major asexual blood stage antigen that is currently considered a strong vaccine candidate. Second, the circumsporozoite protein (CSP), a component of sporozoites that contains a B-cell epitope.

**Methods:**

A synthetic chimeric recombinant 516 bp gene encoding containing PvMSP-1, a Pro-Gly linker motif, and PvCSP was synthesized; the gene, named MLC, encoded a total of 172 amino acids. The recombinant gene was modified with regard to codon usage to optimize gene expression in *Brassica napus*. The Ti plasmid inducible gene transfer system was used for *MLC *chimeric recombinant gene expression in *B. napus*. Gene expression was confirmed by polymerase chain reaction (PCR), beta-glucuronidase reporter gene (GUS) assay, and Western blot.

**Results:**

The MLC chimeric recombinant protein expressed in *B. napus *had a molecular weight of approximately 25 kDa. It exhibited a clinical sensitivity of 84.21% (n = 38) and a clinical specificity of 100% (n = 24) as assessed by enzyme-linked immunosorbent assay (ELISA). Oral immunization of BALB/c mice with MLC chimeric recombinant protein successfully induced antigen-specific IgG1 production. Additionally, the Th1-related cytokines IL-12 (p40), TNF, and IFN-γ were significantly increased in the spleens of the BALB/c mice.

**Conclusions:**

The chimeric MLC recombinant protein produced in *B. napus *has potential as both as an antigen for diagnosis and as a valuable vaccine candidate for oral immunization against vivax malaria.

## Background

*Plasmodium vivax*, a causative agent of relapsing benign tertian malaria, is the second most important malaria-causing species; it afflicts several hundred million people annually [1,2]. Malaria constitutes a major health problem and is closely associated with socioeconomic burden in many temperate and most tropical countries. The malaria situation of Korea peninsula is also not different from other countries. It reemerged in the early 1990s after two decade-long absence. Following government intervention, reported cases of malaria decreased over the course of several years. However, it is unlikely that malaria has been completely eradicated from Korea; not only is there a steady influx of travelers and workers from countries where malaria is endemic, but the appearance of failed treatment cases is also possible. Therefore, vaccine development is needed for the eradication of vivax malaria in Korea.

Compared to *Plasmodium falciparum *vaccine development, less progress has been made on the development of a vivax malarial vaccine due to the lower mortality associated with vivax malaria and limitations in culturing *P. vivax in vitro*. A vaccine to prevent vivax malaria is needed not only to prevent the morbidity associated with the disease but also to prevent the potential spread of malaria due to the reactivation of *P. vivax *hypnozoites in non-endemic areas. It is associated with dormant liver stage infections that can reactivate weeks to months after primary infection and symptomatic disease [3-7].

In this study, a multistage plant-based vaccine containing *P. vivax *merozoite surface protein-1 (PvMSP-1), a Pro-Gly linker motif, and *P. vivax *circumsporozoite protein (PvCSP). The MSP-1 gene encodes a 180 to 230 kDa glycoprotein that is a major merozoite surface protein in several *Plasmodium *species. PvMSP-1 has been thoroughly investigated as a vaccine candidate against the asexual blood stage of malaria [8-11]. PvCSP is a sporozoite stage surface antigen. It consists of 18 to 20 short repeated sequences flanked by pre-repeat and post-repeat areas. Across global isolates, the nine amino acid tandem repeats were primarily GDRA(D/A)GQPA for the VK210 subtype [12] and GNGA(A/G)GQAA for the VK247 subtype [13]. CSPs from Korean *P. vivax *isolates were also shown to contain the nine amino acid tandem repeat sequences [14-16]. The region containing the tandem repeats was cloned to make a recombinant protein that has subsequently been used for malaria diagnosis in patients, sero-epidemiological investigations, and the production of monoclonal antibodies. Additionally, this recombinant protein was reported to be a viable vaccine candidate when assessed for immunogenicity [17-19].

Since the introduction of vaccine production using transgenic plants by Mason et al. [20], oral immunogenicity has been demonstrated for several antigens derived from human and animal pathogens expressed in transgenic plants. For example, the hepatitis B surface antigen was expressed in potatoes [21,22], a viral peptide vaccine was expressed in *Arabidopsis *[23], and the MSP antigens of *P. falciparum *and *Plasmodium yoelii *were expressed in tobacco [24,25]. Additionally, oral immunization with edible vaccines that have been produced in transgenic plants may stimulate immune responses against their target pathogens [26,27]. However, commercially available vaccines based on transgenic plants have not yet to be produced.

In this study, the antigenicity of a chimeric recombinant protein expressed in transgenic *Brassica napus *was investigated and evaluated as an edible vaccine candidate against vivax malaria.

## Methods

### Blood sample collection

Patients exhibiting clinical signs of malaria infection at the Public Health Centers in Gimpo-si and Paju-si of Gyeonggi-do, South Korea, were examined for malaria parasites. Approximately 3 ml of blood was collected from each symptomatic patient. Thin and thick blood smears were prepared for microscopic examination (magnification × 1,000). Blood samples were transported to the Korean National Institute of Health (KNIH), where the sera were separated and stored at -20°C for future examination. Informed consent was obtained from all patients. All samples were collected under human use protocols that were reviewed and approved by the Human Ethics Committee of the Korean National Institute of Health (Cheongwon, Korea).

### Gene construction strategy for the chimeric recombinant protein

**Figure 1:**
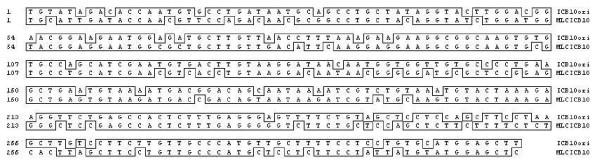
**Nucleotide differences between the original (ICB10ori, GenBank access no**. AF435594**) and the modified (MLCICB10) nucleotide sequences of *P. vivax *MSP-1**.

**Figure 2:**
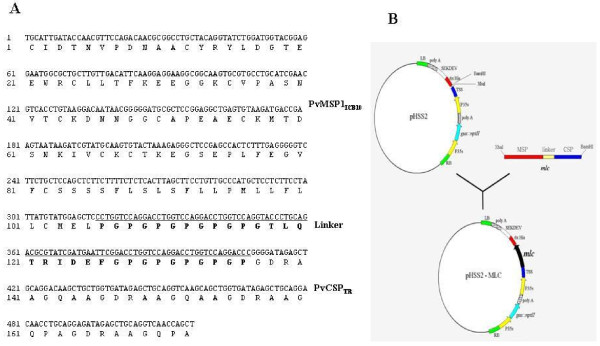
**(A) Gene construction scheme for the expression of the MLC chimeric recombinant protein**. LB and RB indicate the left and right borders of the T-DNA region, which is stably integrated into nuclear chromosomal DNA during *Agrobacterium*-mediated DNA delivery. TSS represents the translation start site, and SEKDEV (Serine-Glutamate-Lysine-Aspartate-Glutamate-Valine) stands for secretion signal. Additionally, this signal promotes exact oligomerization. The selectable marker used for plant transformation is the nptII gene, which confers kanamycin resistance. The GUS reporter protein was used as a marker to enable rapid screening in selective steps of plant regeneration. (B) Nucleotide and deduced amino acid sequence of the MLC chimeric recombinant gene. It is comprised of a modified PvMSP-1 (PvMSP1_ICB10_, AF435594), a linker (Pro-Gly repeat, underlined), and PvCSP (PvCSP_TR_, AF316582).

The chimeric recombinant protein was designed as a biphasic antigen to target both the blood and the early liver cell stages of *P. vivax *infection. One of the epitopes used in the current study was derived from the PvMSP-1 gene of the *P. vivax *Belem isolate; it encompasses nucleotides 4,925 to 5,239 (315 bp), which correspond to the interspecies conserved block 10 (ICB10) (GenBank Access No. AF435594) [28]. The nucleotide sequence of PvMSP-1 gene was modified for optimal expression in plants, as shown in Figure 1. The other epitope was synthesized four repeats of GDRAAGQ(P/A)A for the expression of PvCSP VK210 subtype (AF316582) [14-16,28]. The ICB10 of PvMSP-1 and PvCSP VK210 subtype were linked via Pro-Gly repeat motifs. The linker region contained five or six Pro-Gly repeats surrounding an internal sequence of 10 amino acids, TLQTRIDEFG (Figure 2A) [29,30].

### Construction of plasmids for transgenic plants

To construct the codon-optimized chimeric gene, complementary strands of the oligonucleotides for PvMSP-1, the linker, and PvCSP were synthesized with *Xba*I sites at the 5' end of PvMSP-1 and *Bam*HI sites at the 3' end of PvCSP. These synthesized oligonucleotides were linked by T4 ligase in the order of 5'-PvMSP-1-linker-PvCSP-3' (MLC). This gene was then cloned into the pCR2.1 cloning vector (Invitrogen Co., Carlsbad, CA, USA) subsequently transformed into *Escherichia coli *INVαF' according to the manufacturer's instructions. Resulting transformants were confirmed by digestion with *Eco*RI. Plasmids were then purified and digested with *Xba*I and *Bam*HI restriction enzymes. This gene fragment was then inserted into the protein expression vector pHSS2 vector (Nexgen Co., Seoul, Korea) under the strong constitutive cauliflower mosaic virus 35S (CaMV35S) promoter with a beta-glucuronidase (GUS) reporter gene and a neomycin resistance gene (Figure 2B). The resulting plasmid was amplified in *E. coli *DH5α.

### Transformation, selection, and regeneration of transgenic plants

*Brassica napus *was used as the host plant for transformation. Plant cultivation was performed under greenhouse conditions. Transgenic plants were obtained using the cotyledon agrobacterial transformation technique [31]. To acquire cotyledon explants, seeds were germinated in a culture room at 25°C in a photoperiod of 16 h light/8 h dark for 4 days with the light supplied by cool-white daylight fluorescent lights. The seeding medium contained 0.2% Murashige and Skoog medium with Gamborg vitamin B5 (MSB5) salts, 1% w/v sucrose, 0.05% 2-(N-morpholino) ethane**s**ulfonic acid (MES), and 0.6% plant agar. Cotyledon explants excised from *in vitro *grown seedlings were cut into 0.5 to1 cm pieces, and the apical shoot tip was removed. *Agrobacterium tumefaciens *used for infection and co-cultivation; it was grown in Yeast Extract Peptone (YEP) media (0.5% yeast extract, 0.5% peptone, and 0.5% sucrose, pH 7.2) with kanamycin overnight to an optical density of 1.0 at 600 nm. Cotyledons were immersed in the infection media (37 ml liquid infection medium consisting of 0.4% MSB5 salts, 2% sucrose, 1 μg/ml of 2,4-D, 2 ml dimethyl sulfoxide (DMSO) and a 10 ml pellet of *Agrobacterium *culture) and incubated for 20 min [32]. Excess bacterial culture was removed by blotting with filter paper and the cotyledons were co-cultivated on filter paper-covered co-culture medium (0.05% MES and 0.6% plant agar in infection medium) for two days. Plates were then transferred to dark conditions at 4°C for 2 days. The cotyledons were placed on a selection medium consisting of 0.4% MSB5 salts, 3% w/v sucrose, 0.05% MES, 2 μg/ml 6-benzylaminopurine (BA), 0.01 μg/ml α-naphthalene acetic acid (NAA), and 0.6% plant agar supplemented with 15 μg/ml kanamycin for selective pressure, 500 μg/ml carbenicillin to eliminate the *Agrobacterium *and 2 μg/ml silver nitrate to prevent cell necrosis. The calluses in the selection medium were incubated at 25°C and then transferred to fresh medium every two weeks. The same selection medium was used for shoot regeneration medium. From GUS-positive explants, regenerated shoots that had elongated to 2 to 3 cm in length were transferred to rooting medium. Approximately 6 weeks later, green shoots of approximately 5 to 6 cm in length were isolated and placed in Magenta boxes (Sigma, St. Louis, MO, USA) containing rooting medium and carbenicillin and kanamycin at the same concentrations as shoot regeneration medium. Root initiation was observed within two to three weeks, and roots developed into mature plants within one month.

### GUS (beta-glucuronidase) reporter gene assay

Regenerated shoots were subjected to histochemical analysis for GUS expression. Identification of GUS activity was performed using 5-bromo-4 chloro-3-indolyl β-D-glucuronide (X-Gluc) as a histochemical substrate. For each shoot, 0.1 g was incubated in X-Gluc solution (0.5 mM each of potassium ferro- and ferri-cyanide, 0.3% Triton X-100 and 1.9 mM X-Gluc dissolved in dimethylformamide) at 37°C for 1 h. A GUS-positive response was revealed by a green-blue-colored solution, whereas a negative signal was indicated by a yellow-coloured solution. GUS activity was measured by comparing the optical densities of wild type plants (used as a negative control), transgenic plants with empty vector pHSS2 (used as a positive control) and transgenic plants with pHSS2-MLC as the target product.

### DNA extraction from transgenic plants

For DNA extraction, 200 mg of leaves were homogenized in 400 μl of extraction buffer containing 0.2 mM Tris-HCl (pH 7.5), 250 mM NaCl, 25 mM ethylenediaminetetraacetic acid (EDTA), and 0.5% sodium dodecyl sulfate (SDS). Mixtures were transferred to 1.5 ml tubes that were then agitated and centrifuged. Supernatants were collected and combined with 600 μl chloroform:isoamyl alcohol (24:1, v/v) and vortexed. After centrifugation, supernatants were precipitated with an equal volume of isopropanol; pellets were washed twice with 70% ethanol. The washed pellets were then dissolved in 20 μl TE buffer (10 mM Tris-HCl, 1 mM EDTA, pH 8.0). DNA samples were verified by electrophoresis on a 1% agarose gel. The extracted plant genomic DNA was used as a template for polymerase chain reaction (PCR).

### PCR analysis and sequencing of MLC and nptII genes

Regenerated plants transformed with *A. tumefaciens *GV3101 harbouring the pHSS2 plasmid containing the nptII and MLC genes were identified by PCR analysis. PCR analysis was performed in reaction mixtures containing approximately 0.5 to 1 μg genomic plant DNA under the following conditions: 94°C for 1 min, 30 cycles of 94°C for 1 min, 62°C for 1 min and 72°C for 1 min, and a final extension of 72°C for 10 min in a TECHNE PCR thermocycler (Burlington, NJ, USA). The 516-bp and 800-bp PCR products were obtained using primer sets specific for the MLC gene (MLC-F, 5'-TTCTAGAGTGCATTGATACCAACGTTCC-3' and MLC-R, 5'-TAGGATCCCCAGCTGGTTGACC-3') and nptII gene (NPTII-F, 5'-GAACAAGATGGATTGCACGC-3' and NPTII-R, 5'-GAAGAACTCGTCAAGAAGGC-3'), respectively. To confirm nucleotide sequences, plasmid DNA of *A. tumefaciens *GV3101 (pHSS2-MLC) and the PCR products of the transgenic plant genomic DNA samples were analyzed by Bionics Inc. (Seoul, Korea).

### Preparation of plant protein extracts for immunoblot analysis

To prepare plant protein extracts for enzyme-linked immnunosorbent assay and western blot analysis, plant tissue samples were frozen in liquid nitrogen, ground, and homogenized in extraction buffer [10% trichloroacetic acid (TCA) in acetone with 2% β-mercaptoethanol] [33]. For 1 g of fresh plant tissue, 3 ml buffer was used. Extracts were placed at -20°C for 30 min and then centrifuged at 7,000 × g for 30 min at 4°C. Supernatants were discarded, and the remaining white pellet was washed twice with 10 ml acetone. Plant protein extracts were separated by sodium dodecyl sulfate-polyacrylamide gel electrophoresis (SDS-PAGE), and proteins were transferred onto polyvinylidene fluoride (PVDF) membranes using a semi-dry transfer method (Bio-RAD, Hercules, CA, USA). For western blot analysis, membranes were probed with antibodies directed against PvMSP-1 (Joongkyem Antibody therapeutics, Shiheung, Korea), or PvCSP (supported by Wirtz RA, CDC, USA). Wild type and pHSS2 empty vector-inserted transgenic plants were used as negative controls.

### Immunization of mice with MLC chimeric recombinant protein

Fifteen male BALB/c mice, aged six weeks (Orient Bio Inc, Seongnam, Korea), were divided into three groups. Group A mice were fed 1 g of ground leaves from non-transgenic *B. napus *dissolved in 1.5 ml PBS. Group B mice were fed 200 μg of ground leaves from MLC-transgenic *B. napus*. Group C mice were fed 1 g of ground leaves from MLC-transgenic *B. napus*. All mice were provided food and water *ad libitum*. However, mice were fasted overnight before oral administration of the recombinant protein. Oral administration was performed twice at four-week intervals. Three weeks after the last oral feeding, mice were sacrificed and spleen and blood samples were taken. Sera were analyzed by ELISA and by cytokine and immunoglobulin sub-typing assays. All mouse experiments were performed following protocols approved by the Kyung Hee University Institutional Animal Care and Use Committee.

### Enzyme-linked immunosorbent assays

Sera from patients infected with *P. vivax *were analyzed for the presence of antigen-specific antibodies using 96-well plates coated overnight at 4°C with 0.5 μg/ml per well purified MLC chimeric recombinant protein dissolved in phosphate buffer saline (PBS), pH 7.4. Patient serum samples were diluted 1:100 (v/v) in blocking buffer (0.25% PBS-Tween 20 with 1% bovine serum albumin, pH 7.4) added to the plates and incubated for 1 h. After washing the wells with a 0.05% PBS-Tween 20 solution three times, plates were incubated with peroxidase-conjugated goat anti-human IgG secondary antibody diluted 1:1,000 (v/v, Sigma). For color development, 100 μL of 2.2'-azino-di-(3-ethyl-benzthiozoline-6-sulfonic acid) (ABTS) peroxidase substrate (Kirkegaard & Perry Laboratories, Gaithersburg, MD, USA) was added to each well and incubated at room temperature for 30 min. The absorbance of the mixture was measured at 405 nm (Molecular Devices, Sunnyvale, CA, USA). The cut-off value was taken as the mean ± 2 standard deviations of the negative samples.

### Cytokine and immunoglobulin assays

Spleens from orally immunized mice were minced with a razor and added to 1 ml ice cold endotoxin-free PBS containing a protease inhibitor cocktail (Roche) and 0.1% Igepal CA-630 nonionic detergent (Sigma) [34]. Spleen lysates were then centrifuged at 20,000 × g for 10 min. Supernatants were collected and stored at -20°C for cytokine analysis. The presence of IL-2, IL-12(p40), IFN-γ, and TNF in spleen lysates was determined using Bio-Plex Kits (Bio-Rad) according to the manufacturer's protocols. The results were analyzed using Bio-Plex Manager 5.0 software (Bio-Rad). To analyze the isotype of the antibodies produced by oral immunization, the mouse immunoglobulin isotyping kit (Millipore, Billerica, MA, USA) was used. Briefly, all sera collected 3 weeks after the final immunization were diluted with the MILLIPLEX MAP Assay Buffer at 1:25,000 (v/v). The serum concentrations of each antibody isotype (IgG1, IgG2a, IgG2b, IgG3, IgA, and IgM) were measured by Luminex 100™ IS (Luminex, Austin, TX, USA).

### Statistical analysis

All data are presented as the means ± standard deviation; results were statistically analyzed. IL-2, IL-12 (p40), IFN-γ, and TNF levels among the three groups were compared using one-way ANOVA followed by Bonferroni's multiple comparison tests. A *P*-value of < 0.05 was considered to be statistically significant.

## Results

### Construction of an MLC chimeric recombinant protein

The MLC chimeric recombinant gene was synthesized using the ICB10 of the *P. vivax *MSP-1 gene and nine tandem repeats from the *P. vivax *VK210 subtype CSP gene. The PvMSP-1 gene was modified to optimize gene expression in plants without changing amino acid codon usages as shown in Figure 1. Following optimization, the synthetic PvMSP-1 gene sequence was revealed to have 75.6% identity with the Belem isolate of *P. vivax *(Figure 1). The linker region contained six Pro-Gly repeats before and five after an internal sequence of 10 amino acids, 'TLQTRIDEFG' (Figure 2A). The last G of the Pro-Gly repeat overlapped with the start of the CSP repeats. The repeat sequence of PvCSP was added after the linker region with two 'GDGAAGQAA' and two 'GDRAAGQPA' sequences. The resulting synthetic MLC chimeric recombinant gene, named the MLC gene, was composed of 105 amino acids from PvMSP-1, a 31-amino-acid linker, and 36 amino acids from tandem repeats of PvCSP VK210 subtype (Figure 2A).

### Transformation of the MLC chimeric recombinant gene into *B. napus*

**Figure 3:**
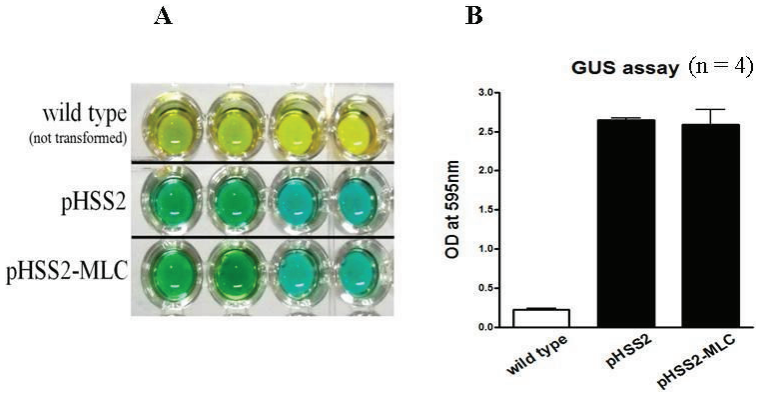
**Detection of GUS activity in explants of regenerated *B. napus *plants**. (A) GUS reporter activity as measured by color change. (B) GUS reporter activity was quantified by measuring sample optical densities at 595 nm.

**Figure 4:**
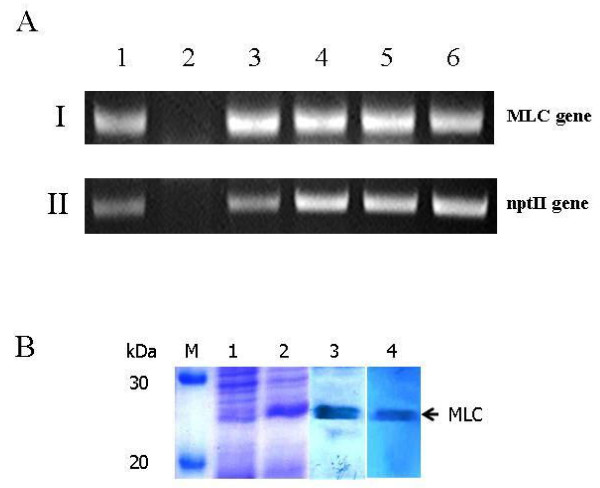
**(A) Confirmation of transformed *B. napus *by PCR amplification of the MLC gene (I) and the nptII gene (II)**. Lanes 1, 3-6, transformed explants; Lane 2, non-transformed explant. (B) Expression and confirmation of the MLC chimeric recombinant protein in *B. napus*. Lane 1, wild type plant; Lane 2, pHSS2-MLC transgenic plant; Lane 3, reacted with anti-PvMSP antibodies; Lane 4, reacted with anti-PvCSP antibodies.

The MLC chimeric recombinant gene was inserted into the pHSS2 vector at *Xba*I and *Bam*HI sites for expression in *B. napus*. The resulting expression vector was referred to as pHSS2-MLC. It was transformed into *B. napus *using an *Agrobacterium*-mediated DNA delivery system (Figure 2B). Three transgenic plant groups were constructed: Group I, non-transgenic plants (wild type); Group II, plants transformed with *A. tumefaciens *GV3101/pHSS2 empty plasmid (negative control), and Group III, plants transformed with *A. tumefaciens *GV3101/pHSS2-MLC. Non-transgenic plants showed a yellow color in the GUS assay (Figure 3A), with an optical density of 0.23 ± 0.02 (n = 4, Figure 3B). *B. napus *plants transformed with the *A. tumefaciens *GV3101/pHSS2 plasmid produced 85 calluses, 62 of which exhibited a green spot in the GUS reporter gene assay (Figure 3A), with an optical density of 2.66 ± 0.05 (n = 4, Figure 3B). These results indicated the presence of the target plasmid in plants. Of the 32 calluses obtained from the transgenic *B. napus *plants with *A. tumefaciens *GV3101/pHSS2-MLC, 23 showed a positive response in the GUS reporter gene assay (Figure 3A), with an optical density of 2.59 ± 0.16 (n = 4, Figure 3B). Up to 90% of the GUS reporter gene-expressing plants were positive when screened for the presence of the recombinant gene by PCR analysis. The PCR products amplified with MLC specific primer consistently included the expected 550-bp band in MLC transformed plants, while PCR on the non-transformed plants failed to amplify the target MLC gene. PCR analysis using an nptII-specific primer set showed products of approximately 800 bp (Figure 4A). Further, DNA sequencing analysis of genomic DNA from transgenic *B. napus *plants demonstrated identical sequences to the MLC chimeric recombinant gene.

### Immune response to MLC chimeric recombinant protein in BALB/c mice

**Figure 5:**
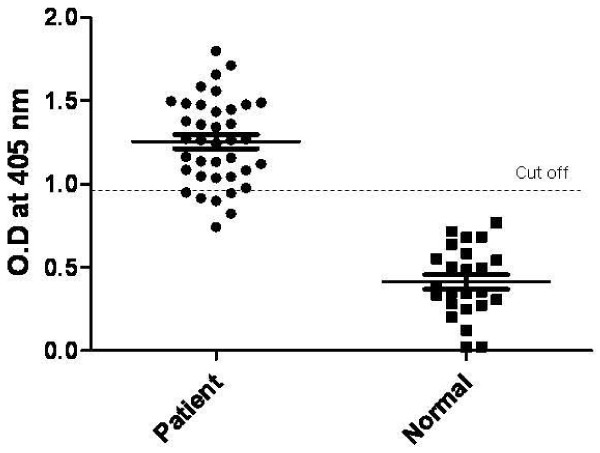
**Scattergram of absorbances measured by ELISA using MLC chimeric recombinant protein expressed in *B. napus***. Sera from healthy individuals and malaria patients infected with *P. vivax *were used.

To investigate the antigenicity of the MLC chimeric recombinant protein expressed in *B. napus*, the protein was purified using the TCA-acetone-based protein precipitation method. SDS-PAGE analysis determined the molecular mass of the protein to be approximately 25 kDa (Figure 4B. lane 2). As shown in Figure 4B, the recombinant protein reacted with anti-PvMSP-1 (lane 3) and anti-PvCSP antibodies (lane 4). The protein exhibited a clinical sensitivity of 84.21% (n = 38) and 100% specificity (n = 24) in ELISA (Figure 5). Therefore, the MLC chimeric recombinant protein displayed antigenic properties *in vitro*.

**Figure 6:**
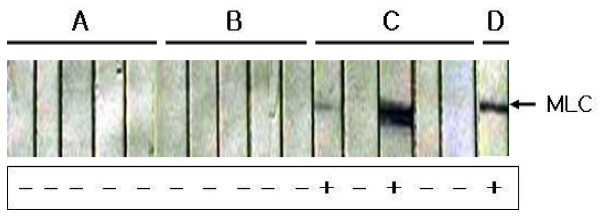
**Antibody induction in BALB/c mice orally immunized with transgenic *B. napus *that expresses the MLC chimeric recombinant protein**. (A) BALB/c mice fed PBS only; (B), BALB/c mice fed 200 μg of chimeric recombinant protein; (C), BALB/c mice fed 1 g of chimeric recombinant protein; (D), positive control-reacted with anti-his antibody.

**Figure 7:**
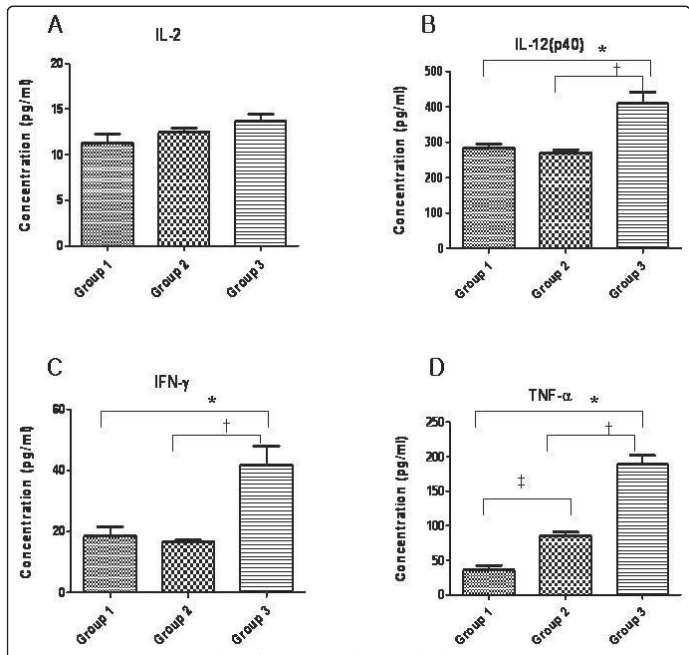
**Cytokine expression in the spleens of BALB/c mice orally immunized with transgenic *B. napus *that expresses the MLC chimeric recombinant protein**. Concentrations of IL-2, IL-12(p40), IFN-γ, and TNF-α were measured. Group 1, BALB/c mice fed PBS only; Group 2, BALB/c mice fed non-transformed plant; Group 3, BALB/c mice fed MLC chimeric recombinant protein. Values represent the mean ± SD (n = 3). * Significant difference between Groups 1 and 3 (*P *< 0.05). ^†^Significant difference between Groups 2 and 3 (*P *< 0.05).

Serum samples were collected from each group of mice three weeks after the final immunization and tested for MLC chimeric recombinant protein-specific antibodies using Western blot analysis. Antigen-specific antibodies were not detected in PBS fed mice (Figure 6A) or in mice fed 200 μg of chimeric recombinant protein (Figure 6B). However, two of the five mice fed 1 μg of MLC chimeric recombinant protein showed a positive reaction by Western blot (Figure 6C). In an isotype analysis, IgG1 levels increased in the sera of BALB/c mice fed with extract of pHSS2-MLC transformed *B. napus*. The mean amount of IL-2 expression in spleen was 11.28 ± 1.66 pg/ml in Group 1 mice, which were fed only PBS, 12.44 ± 0.89 pg/ml in Group 2 mice, fed *B. napus *transformed with empty expression vector only (pHSS2), and 13.68 ± 1.38 pg/ml in Group 3 mice, which were fed *B. napus *that expressed the MLC chimeric recombinant protein. There were no significant differences between groups (Figure 7A). The mean amount of IL-12(p40) expression was 283.97 ± 19.75 pg/ml in Group 1, 270.54 ± 14.65 pg/ml in Group 2, and 410.77 ± 54.58 pg/ml in Group 3. There was a significant difference between Groups 1 and 3, and between Groups 2 and 3, but there was no statistically significant difference between Groups 1 and 2 (*P *< 0.05) (Figure 7B). The mean amount of IFN-γ expression was 18.50 ± 5.53 pg/ml in Group 1, 16.61 ± 0.98 pg/ml in Group 2, 41.80 ± 10.88 pg/ml in Group 3. There was a significant difference between Groups 1 and 3, and between Groups 2 and 3, but there was no statistically significant difference between Groups 1 and 2 (*P *< 0.05) (Figure 7C). The mean amount of TNF-α expression was 35.56 ± 11.94 pg/ml in Group 1, 85.98 ± 7.90 pg/ml in Group 2, and 188.90 ± 22.74 pg/ml in Group 3. There was a significant difference between Groups 1 and 3, between Groups 2 and 3, and between Groups 1 and 2 (*P *< 0.05) (Figure 7D). Therefore, the Th1-related cytokines, IL-12(p40), IFN-γ, and TNF were expressed in the spleens of BALB/c mice orally immunized with ground leaves from transgenic plants that expressed the MLC chimeric recombinant protein.

## Discussion

A number of malarial proteins that have been demonstrated to be potential vaccine candidates are currently in various stages of pre-clinical and clinical trials [35-37]. Many of these trials are based on MSP, CSP, and apical membrane antigen-1 (AMA-1) proteins [38-41]. A vast majority of the vaccine candidates target *P. falciparum*, with fewer *P. vivax *candidates in vaccine development [42]. Presently, there are only two *P. vivax *subunit vaccine candidates undergoing clinical development and only a modest number of other candidates in pre-clinical trials [5]. Additionally, there are several trials of chimeric malaria vaccines constructed using MSP-8/MSP-1 and AMA-1/MSP-1. However, although these chimeric vaccines successfully induced high antibody responses, they failed to protect against infectious challenge with parasites [29,37,43].

In the present study, an MLC chimeric recombinant protein was developed which containing the ICB10 region of MSP-1 and four tandemly repeated sequences of nine amino acids from CSP VK210 subtype. The strategy of gene construction was the first challenge in making an MLC chimeric recombinant protein for a multistage combination vaccine directed against both merozoite and sporozoite forms. *B. napus *was selected for the expression of an MLC chimeric recombinant gene. The expression and purification of recombinant proteins in plants has several advantages compared to other commercial expression systems. First, plant-based vaccines do not contain endotoxin, which is produced during bacterial culture and purification steps. Additionally, the biomass produced in plants is greater than other systems, thereby reducing the cost and allowing rapid industrialization and manufacturing [22,23]. Also, if edible plants are utilized, plant-based vaccines can be eaten raw without modification in factories. Finally, expression of candidate vaccine proteins in plants allows for the complete synthesis of target antigens that are able to induce an active immune response in animals. In light of these advantages, transgenic plants are now being generated and investigated for use in the production of vaccines against various human pathogens [20,21,24,26].

This study employed classical techniques for the generation of transgenic *B. napus *to produce a transgenic plant containing the MLC chimeric recombinant protein gene under the control of the constitutive CaMV35S promoter [21]. Additionally, the nucleotide sequence of ICB10 region of MSP-1 was modified to optimize its expression in plants (Figure 1). The modified sequence has 75.6% nucleotide sequence homology and 100% amino acid sequence homology compared to the original ICB10 sequence from the *P. vivax *Belem isolate; further, it was successfully expressed in *B. napus *(Figure 4B, lane 2). Purified MLC chimeric recombinant protein reacted well with the sera of vivax malaria patients (Figure 5). Therefore, this reagent could be used to sero-diagnose patient with vivax malaria. Although not all of the mice orally immunized with 1 g MLC chimeric recombinant protein produced antigen-specific antibodies, MLC chimeric recombinant protein-specific IgG1 antibodies were detected in two (40%) of the five mice (Figure 6). These observations indicate that the MLC chimeric recombinant protein has the possibility to trigger Th-2 immune responses. Il-12(p40), TNF, and IFN-γ were detected in the spleens of BALB/c mice orally immunized with the MLC chimeric recombinant protein (Figure 7). However, IL-2 was not produced in significant amounts. Together with these results, MLC chimeric recombinant protein induced both Th-1 and Th-2 immune responses in BALB/c mice effectively.

## Conclusions

Based on this work, the MLC chimeric recombinant protein produced in transgenic *B. napus *plants has potential as a *P. vivax *vaccine candidate. There are several merits unique to *B. napus *for the production of anti-malarial vaccines, including the ease of cultivation of *B. napus *even in the absence of certain nutrients; together, these advantages allow for rapid industrialization and manufacturing. Additionally, because of the convenience of ingesting this plant, populations can be given the vaccine as part of a routine diet. This strategy will decrease the cost of vaccination against malaria in developing countries. Additionally, trials for developing edible plant vaccines will contribute to a basic understanding of the development of vivax malaria vaccines. In conclusion, the MLC chimeric recombinant protein might be helpful in the serodiagnosis and vaccine development against *P. vivax*.

## Competing interests

The authors declare that they have no competing interests.

## Authors' contributions

CL, HHK, SJL, and HWL conceived, designed and contributed to the execution of the study and research. SJL and HWL wrote the manuscript. KMC, KWC, YKC, MJJ, and TSK collected the blood samples in the field. NJC, HGL, HSL, YS, and HK performed PCR, Western blot, ELISA, cytokine, and immunoglobulin assays. HGR, HSL, and NJC helped in the design of the study. Plant engineering was performed by KWC, MJJ, and CL. Mouse experiments were performed by CL, HHK, KMC, and YKC. All authors have read and approved the final manuscript.
